# MED36/451: Health-Related World Wide Web Tool: An environment for building WWW-based healthy community

**DOI:** 10.2196/jmir.1.suppl1.e73

**Published:** 1999-09-19

**Authors:** S Linn

**Affiliations:** ^1^Rambam Hospital and Technion, HaifaIsrael

**Keywords:** Web-tools, Internet

## Abstract

**Introduction:**

It is difficult for doctors that lack technical background to create WWW-based community of patients and doctors.

**Methods:**

Interviews with physicians and medical students. Qualitative analysis.

Results and Discussion:

There is a need for easy-to-use tools for creating WWW-based health -related environment that are otherwise beyond the ability of the non-computer programmer. These tools should not only produces web pages for physicians the on the WWW, but also create web-based communities that allow sharing of information between health professionals as well as between health professionals and the public. Examples of tools would include bulletin boards, patient's forums, health self-evaluation, electronic mail, administrative information such as clinics hours, procedures and payment schedules, and links to other major medical sites. There should be no requirements that each physician set up and maintain their own server. These servers will be maintained centrally in an organization (such as a hospital) and will be used by members of that organization. The physician should not be required to have any knowledge of HTTP at all. These tools should be universal and compatible with any server.

**Conclusion:**

This tool could be extremely helpful and succesful.

